# Analysis of complete chloroplast genome sequences and insight into the phylogenetic relationships of *Ferula* L

**DOI:** 10.1186/s12864-022-08868-z

**Published:** 2022-09-08

**Authors:** Lei Yang, Ozodbek Abduraimov, Komiljon Tojibaev, Khabibullo Shomurodov, Yuan-Ming Zhang, Wen-Jun Li

**Affiliations:** 1 State Key Laboratory of Desert and Oasis Ecology, Xinjiang Institute of Ecology and Geography, Chinese Academy of Sciences, No.818 South Beijing Road, Urumqi, 830011 China; 2 Xinjiang Key Lab of Conservation and Utilization of Plant Gene Resources, No.818 South Beijing Road, Urumqi, 830011 China; 3 College of Resources and Environment, University of Chinese Academy of Sciences, Shijingshan District, No.19(A) Yuquan Road, Beijing, 100049 China; 4 Institute of Botany, Uzbekistan Academy of Sciences, No.32 Durmon Yuli Street, Tashkent, Uzbekistan 100125; 5 Sino-Tajikistan Joint Laboratory for Conservation and Utilization of Biological Resources, No.818 South Beijing Road, Urumqi, 830011 China; 6 The Specimen Museum of Xinjiang Institute of Ecology and Geography, Chinese Academy of Sciences, Urumqi, 830011 China

**Keywords:** *Ferula*, Chloroplast genome, Comparative analysis, Phylogenetic relationships

## Abstract

**Background:**

*Ferula* L. is one of the largest and most taxonomically complicated genera as well as being an important medicinal plant resource in the family Apiaceae. To investigate the plastome features and phylogenetic relationships of *Ferula* and its neighboring genera *Soranthus* Ledeb., *Schumannia* Kuntze., and *Talassia* Korovin, we sequenced 14 complete plastomes of 12 species.

**Results:**

The size of the 14 complete chloroplast genomes ranged from 165,607 to 167,013 base pairs (bp) encoding 132 distinct genes (87 protein-coding, 37 tRNA, and 8 rRNA genes), and showed a typical quadripartite structure with a pair of inverted repeats (IR) regions. Based on comparative analysis, we found that the 14 plastomes were similar in codon usage, repeat sequence, simple sequence repeats (SSRs), and IR borders, and had significant collinearity. Based on our phylogenetic analyses, *Soranthus*, *Schumannia*, and *Talassia* should be considered synonymous with *Ferula*. Six highly divergent regions (*rps16*/*trnQ-UUG*, *trnS-UGA*/*psbZ*, *psbH*/*petB*, *ycf1*/*ndhF*, *rpl32*, and *ycf1*) were also detected, which may represent potential molecular markers, and combined with selective pressure analysis, the weak positive selection gene *ccsA* may be a discriminating DNA barcode for *Ferula* species.

**Conclusion:**

Plastids contain abundant informative sites for resolving phylogenetic relationships. Combined with previous studies, we suggest that there is still much room for improvement in the classification of *Ferula*. Overall, our study provides new insights into the plastome evolution, phylogeny, and taxonomy of this genus.

**Supplementary Information:**

The online version contains supplementary material available at 10.1186/s12864-022-08868-z.

## Background

*Ferula* L., a perennial single- or multi-bearing herb in the family Apiaceae, contains approximately 170 species mainly distributed in the Mediterranean region of southern Europe, northern Africa, Iran, Afghanistan, Central Asia, Siberia, Russia, India, and Pakistan [1]. Some *Ferula* species can secrete aromatic resins that have the aroma of onions and garlic, and these aromatic resins have insecticidal and fatigue-reducing properties, can be used to treat stomach diseases, dyspepsia, and abdominal pain, and is a plant resource with potentially important medicinal value [2–5].

Due to the similar morphologies and wide distribution of its constituent species, *Ferula* is recognized as one of the most taxonomically complicated genera within the Apiaceae [5–8]. *Ferula* was originally divided into three sections, *Euferula* Boiss., *Peucedanoides* Boiss., and *Scorodosma* Bunge [9], and later into four subgenera, *Scorodosma* (Bunge) Boiss., *Narthex* (Falc.) Drude, *Soranthus* Ledeb., and *Euryangium* (Kauffm.) Drude [10]. Fifty years later, Korovin [11] systematically divided the genus into six subgenera according to fruit, inflorescence, petals, and the number of vitta in fruits, namely *Scorodosma* (Bunge) Drude, *Merwia* (B. Fedtsch.) Korovin (including the sections *Saprosmia* Korovin, *Phacocarpa* Korovin, and *Discicarpa* Korovin), *Narthex* (Falc.) Drude (including the sections *Paleonarthex* Korovin and *Neonarthex* Korovin), *Euferula* (Boiss.) Korovin (including the sections *Phyllites* Korovin and *Anatriches* Korovin), *Peucedanoides* (Boiss.) Korovin (including the sections *Xeronarthex* Korovin and *Macrorrhiza* Korovin), and *Dorematoides* (Rgl. et Schmalh.) Korovin [11]. This arrangement was met with both approval [12] and opposition [13–15]. Safina and Pimenov [8] suggested that the genus *Merwia* was not naturally monophyletic and should be reduced as a section. Integrating the available research, a new classification system was subsequently proposed based on the nuclear ribosomal (nr) DNA internal transcribed spacer (ITS) and three plastid regions (*rps16* intron, *rpoC1* intron, and *rpoB-trnC*) with a total of four subgenera, namely *Sinoferula* Spalik, Puchałka & M.Panahi, *Safinia* Spalik, M.Panahi & Puchałka, *Ferula* (including the sections *Ferula* and *Stenocarpa* Puchałka & Spalik), and *Narthex* (Falc.) Drude (including the sections *Glaucoselinum* (Schischk.) Pimenov, *Macrorrhiza* Korovin, *Soranthus* (Ledeb.) Pimenov, *Peucedanoides* Boiss., *Pachycarpa* (Korovin) Banasiak, *Euryangium* (Kauffm.) Pimenov, *Scorodosma* (Bunge) Boiss., and *Merwia* (B. Fedtsch.) Koso-Pol.) [16]. However, this system still has many problems, such as the incongruence between nrDNA and plastid DNA as well as the chaotic interspecific relationship within the sections *Merwia*, *Scorodosma*, and *Peucedanoides*.

Moreover, the relationship between *Ferula* and some neighboring genera has been debated frequently, especially in the cases of *Soranthus* Ledeb., *Schumannia* Kuntz., and *Talassia* Korovin. *Soranthus* was established as a monotypic genus by Ledebour [17], with *S. sibiricus* (Willd.) Koso-Pol. considered a combination based on *F. sibirica* Willd. as published in 1798. However, this taxonomic treatment was not accepted, and *Soranthus* was subsequently merged into *Ferula* by Bunge [18], Drude [10], Safina and Pimenov [14], and Piminov [1]. Of specific note, *Soranthus* is recognized as a separate genus in the Flora of the Soviet Union [19], the Flora of China [20], and the Flora Xinjiangensis [21]. The same situation occurs in *Schumannia*, which was established as a monotypic genus with the type *S. turcomanica* Kuntz. [22]. *S. turcomanica* is a later homonym of *Ferula karelinii* Bunge, published by Bunge in 1851*.* In 1947, Korovin described the replacement *Schumannia karelinii* (Bunge) Korovin; however, *Ferula karelinii* was also listed within *Ferula* by Bunge [18], Drude [10], Safina and Pimenov [14], Piminov [1], and Tojibaev et al. [23], but not in the Flora of the Soviet Union [19], the Flora of China [20], or the Flora Xinjiangensis [21]. *Talassia renardii* (Regel & Schmalh.) Korovin and *T. transiliensis* (Herder) Korovin, which were isolated from *Peucedanum transiliensis* Regel & Herder from the genus *Peucedanum* L. [24], were recorded in the Flora of Kazakhstan and subsequently transferred to *Ferula* by Pimenov [25] and admitted by Govaerts et al. [26]. However, *Talassia* has also been listed as an independent genus in some Chinese floras [20, 21]. In addition, some studies have suggested that *Schumannia* should be merged with *Soranthus* based on their fruit, pollen morphology, and serological investigations [27, 28]. Recentlly, some molecular phylogeny based on the relatively limited number of nrDNA and cpDNA sequences indicated that *Soranthus*, *Schumannia*, and *Talassia* were embedded in *Ferula*, but show low support values [7, 16, 29].

Chloroplasts are independent organelles in plant cells that have their own complete set of genomes and typically covalently closed circular DNA, which exists in cells as multiple copies [30]. The chloroplast genomes of higher plants have a highly conserved tetrad structure involving inverted repeat sequences (IRs) and large single-copy (LSC) and small single-copy (SSC) regions [31]. Chloroplast genomes are relatively conserved in terms of gene number and sequence in terrestrial plants [32]. The sizes of chloroplast genomes are generally within the range of 115–165 kb, and genome size variation is mainly affected by reverse repeat length variation. Additionally, chloroplast genomes usually exhibit uniparental inheritance and low nucleotide substitution rates [33]. At present, chloroplast genome sequences and nuclear genome sequences can be obtained using shallow whole genome sequencing technology. This is considered an effective means of improving the rate of species identification and has been developed as a tool for plant phylogenetic studies at different taxonomic levels [34–42]. For example, the complete plastomes and nrDNA sequences obtained based on shallow genome sequencing have greatly improved the species identification rate of *Rhododendro*n, which is also difficult to classify [43]. Thus, the complete plastomes might insight into the phylogenetic relationships of *Ferula* and its neighboring genera.

Here, we used plastomes to infer the phylogenetic relationships between *Ferula* and its confused neighboring genera. Fourteen newly sequenced plastomes of *Ferula* (including *Soranthus*, *Schumannia*, and *Talassia*) were analyzed to (1) conduct comprehensive research on the *Ferula* chloroplast genome; (2) identify hotspot regions, microsatellite types, and comparative genomic divergence; (3) analyze the relationships between *Ferula*, *Soranthus*, *Schumannia*, and *Talassia* based on their complete chloroplast genomes; and (4) serve as a reference for subsequent phylogenomic studies of the genus *Ferula*.

## Results

### Chloroplast genome features

**Fig. 1:**
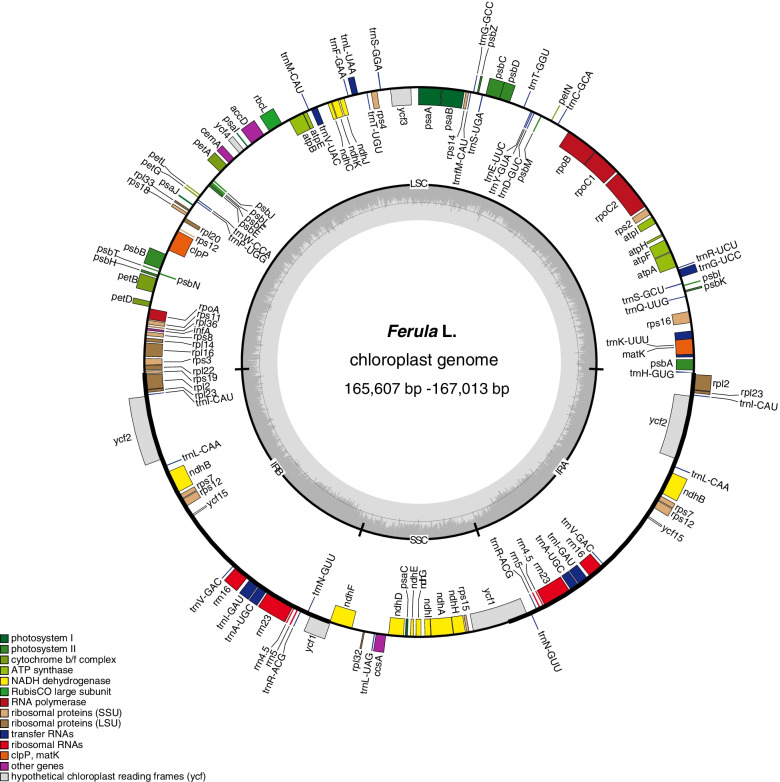
Chloroplast genome maps for *Ferula* L. Genes on the inside of the circle are transcribed clockwise and those on the outside are transcribed counterclockwise. The darker gray inner circle corresponds to the GC content, whereas the lighter gray indicates the AT content. Different colors represent different functional genes

The 14 complete cp genomes ranged from 165,607 to 167,013 bp. Newly sequenced *Ferula* chloroplast genome maps are shown in Fig. 1. All cp genomes possessed the typical quadripartite structure of angiosperms, consisting of a pair of inverted repeat regions (IRs: 31,392–31,880 bp) and a circular molecular structure (Fig. 1; Table 1). All 14 cp genomes possessed 133 distinct genes arranged in the same order, including 87 protein-coding genes, 37 tRNA genes, and eight rRNA genes. Of these, 14 protein-coding genes and eight tRNAs contained at least one intron. The genes were classified into the following four groups based on their functions: (1) 74 self-replication genes; (2) 45 photosynthesis-related genes (in Rubisco, ATP synthase, Photosystem I, cytochrome b/f complex, photosystem II, and NADH dehydrogenase groups); and 13 other genes including (3) six genes with known functions (*matK*, *cemA*, *accD*, *ccsA*, *infA*, and *clpP*) and (4) seven genes with unknown functions (*ycf1*(2), *ycf2*(2), *ycf3*, *ycf4*, and *ycf15*) (Table 2). The total GC content for 12 sequenced species was 37.8–38.0% (Table 1).

**Table 1:** Newly sequenced and complete chloroplast genomes of *Ferula* species

Sample ID	Species	Genome size (bp)	GC content (%)	LSC (bp)	SSC (bp)	IR (bp)
L-6	*F. gigantea*	166,222	37.9	85,383	17,563	31,638
L-12	*F. equisetacea*	165,607	37.9	85,231	17,592	31,392
L-14	*F. sibirica 1*	166,648	37.9	85,346	17,632	31,835
L-15	*F. litwinowiana*	166,554	38	85,226	17,614	31,857
L-23	*F. kelifi*	166,712	38	85,323	17,629	31,880
L-29	*F. transiliensis 1*	166,547	38	85,306	17,599	31,821
L-58	*F. renardii*	166,520	38	85,317	17,559	31,822
L-59	*F. oopoda*	166,565	38	85,328	17,595	31,821
L-60	*F. fedtschenkoana*	166,445	38	85,205	17,568	31,836
L-88	*F. ovina*	166,450	38	85,341	17,561	31,774
L-101	*F. olivacea*	167,013	37.8	85,598	17,687	31,864
L-108	*F. transiliensis 3*	166,520	38	85,293	17,585	31,821
L-109	*F. sibirica 3*	166,644	37.9	85,348	17,626	31,835
L-111	*F. karelinii*	166,037	37.9	84,839	17,592	31,803

**Table 2:** List of genes in the chloroplast genomes of the examined *Ferula* species

Category	Gene group	Gene name
Photosynthesis	Subunits of photosystem I	psaA, psaB, psaC, psaI, psaJ
	Subunits of photosystem II	psbA, psbB, psbC, psbD, psbE, psbF, psbH, psbI, psbJ, psbK, psbL, psbM, psbN, psbT, psbZ
	Subunits of NADH dehydrogenase	ndhA^a^, ndhB^ad^, ndhC, ndhD, ndhE, ndhF, ndhG, ndhH, ndhI, ndhJ, ndhK
	Subunits of cytochrome b/f complex	petA, petB^a^, petD, petG, petL, petN
	Subunits of ATP synthase	atpA, atpB, atpE, atpF^a^, atpH, atpI
	Large subunit of rubisco	rbcL
	Subunits photochlorophyllide reductase	-
Self-replication	Proteins of large ribosomal subunit	rpl14, rpl16^a^, rpl2^ad^, rpl20, rpl22, rpl23^d^, rpl32, rpl33, rpl36
	Proteins of small ribosomal subunit	rps11, rps12^bd^, rps14, rps15, rps16^a^, rps18, rps19, rps2, rps3, rps4, rps7^d^, rps8
	Subunits of RNA polymerase	rpoA, rpoB, rpoC1^a^, rpoC2
	Ribosomal RNAs	rrn16^d^, rrn23^d^, rrn4.5^d^, rrn5^d^
	Transfer RNAs	trnA-UGC^ad^, trnC-GCA, trnD-GUC, trnE-UUC, trnF-GAA, trnG-GCC, trnG-UCC^a^, trnH-GUG, trnI-CAU^d^, trnI-GAU^ad^, trnK-UUU^a^, trnL-CAA^d^, trnL-UAA^a^, trnL-UAG, trnM-CAU, trnN-GUU^d^, trnP-UGG, trnQ-UUG, trnR-ACG^d^, trnR-UCU, trnS-GCU, trnS-GGA, trnS-UGA, trnT-GGU, trnT-UGU, trnV-GAC^d^, trnV-UAC^a^, trnW-CCA, trnY-GUA, trnfM-CAU
Other genes	Maturase	matK
	Protease	clpP^b^
	Envelope membrane protein	cemA
	Acetyl-CoA carboxylase	accD
	c-type cytochrome synthesis gene	ccsA
	Translation initiation factor	infA
	other	-
Genes of unknown function	Conserved hypothetical chloroplast ORF	ycf1, ^c^ycf1, ycf15^d^, ycf2^d^, ycf3^b^, ycf4

Notes: Gene^a^: Gene with one intron

Gene^b^: Gene with two introns

^c^Gene: Pseudo gene

Gene^d^: Number of copies of multi-copy genes

### Codon usage

**Fig. 2:**
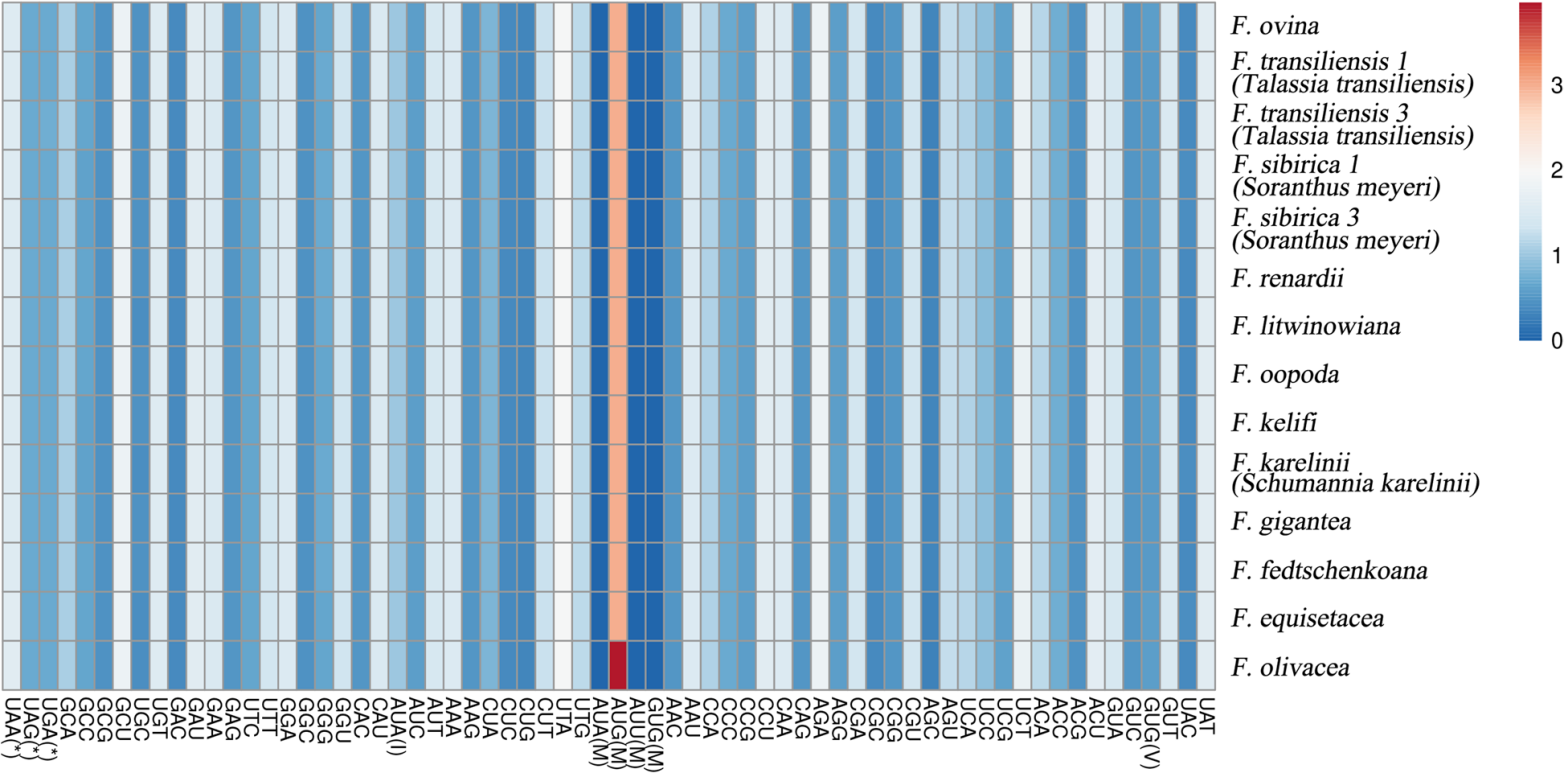
Relative synonymous codon usage (RSCU) values of all merged protein-coding genes for 14 *Ferula* plastomes. Color key: red values indicate higher RSCU values, and blue values indicate lower RSCU values. M = initiation codon, * = termination codon, I = l-isoleucine codon, and V = Valine codon

The RSCU values of all codons are shown in Fig. 2 in the form of a heatmap; the red values indicate higher RSCU values, and the blue values indicate lower RSCU values. For *Ferula* species, the most commonly used transcription initiation codon was AUG, the most commonly used termination codon was UAA, and the initiation codon AUU only existed in *F. olivacea*. Except for the initiation codon and termination codon, the most used transcription codon was UTA, and AGC showed the lowest RSCU values; the most abundant amino acid (AA) was leucine, while cysteine was the lowest frequency AA. Except for tryptophan, all AAs had more than one synonymous codon, and three AAs (leucine, serine, and arginine) had the most (six) synonymous codons. The use of one codon, UGG, showed no bias (RSCU = 1) (Table S2).

### Repeat structure analysis

Forward, palindromic, reverse, and complementary repeats were detected in 14 *Ferula* plastomes. Except for IR repeats, 837 repeats were identified in total; the numbers of forward repeats (398) and palindromic repeats (421) were much higher than the complement repeats (7) and reverse repeats (11). Reverse and complementary repeats were missing in four samples (*F. sibirica* 1, *F. kelifi*, *F. ovina*, and *F. karelinii* 3). *F. kelifi* contained the maximum number of repeats (94), whereas *F. equisetacea* and *F. olivacea* contained the least (46) (Table S3). A total of 1,061 SSRs were identified in the 14 species, six of which did not have pentanucleotides, and hexanucleotides were only found in *F. olivacea*. Additionally, mononucleotides were most frequent followed by dinucleotides, tetranucleotides, trinucleotides, pentanucleotides, and hexanucleotides. *F. transiliensis*-1 contained the highest number of SSRs (82), whereas *F. oopoda* contained the least (69). Poly (A/T) SSRs were typically most common, while poly (C/G) repeats were extremely rare (Table S4).

### Comparisons of border and sequence identity

**Fig. 3:**
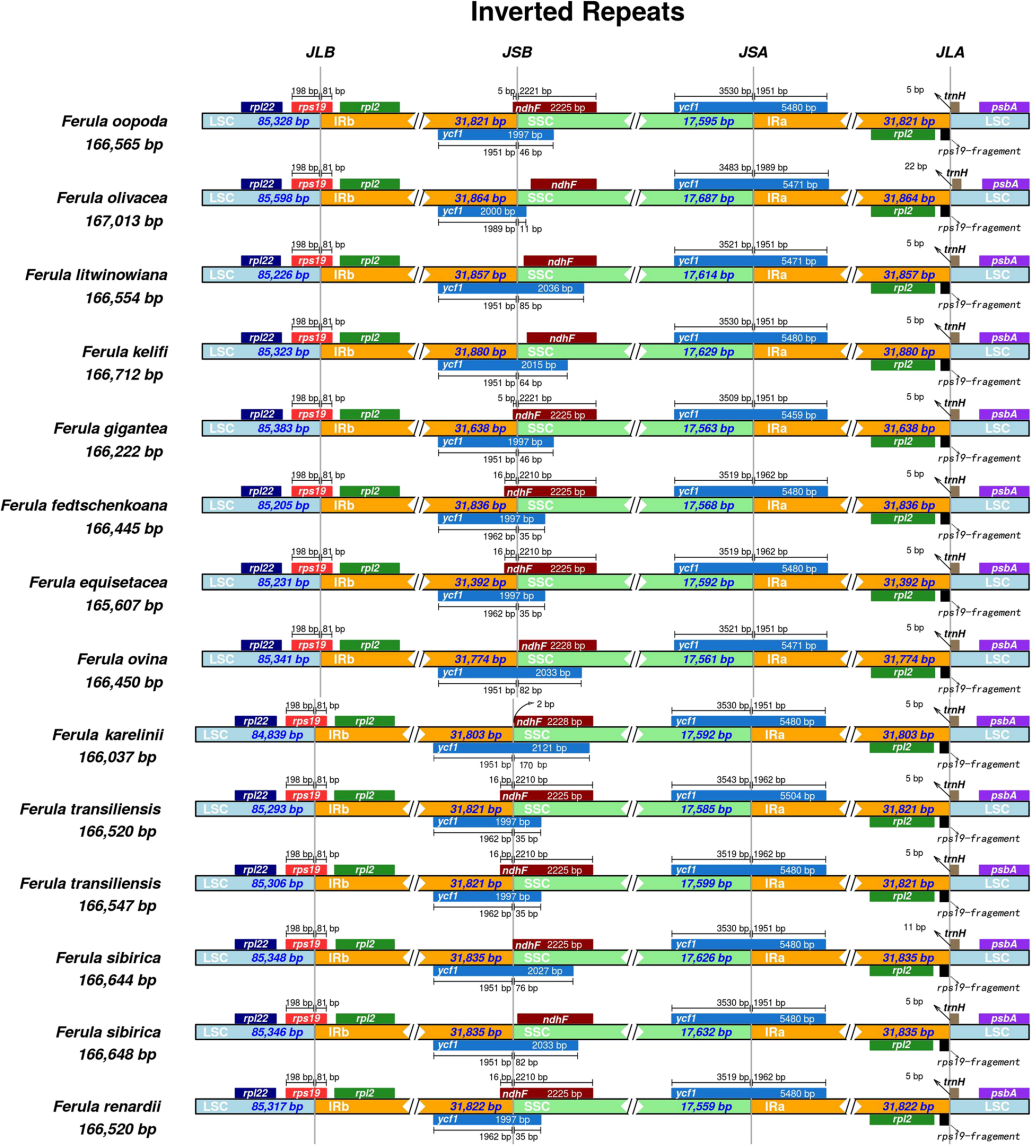
Comparison of the border regions of the 14 studied *Ferula* plastomes

Single-copy and inverted repeat borders were examined; *F. kelifi* and *F. equisetacea* harbored the longest (31,880 bp) and shortest (31,392 bp) IR regions, respectively. Among all 14 *Ferula* species, *rps19* is embedded in the LSC/IRb junction region and only 81 bp with the IRb overlap; *ycf1* spans SSC/IRa and occupies a long section in both regions; and *trnH* occurs in the LSC region and is only 5 bp away from IRa, except for *F. sibirica* 3 (11 bp). The variety of IRb/SSC is relatively high, most (or all) of which occur in the SSC region, and the overlap with the IRb region varied from -18 to 16 bp (Fig. 3).

**Fig. 4:**
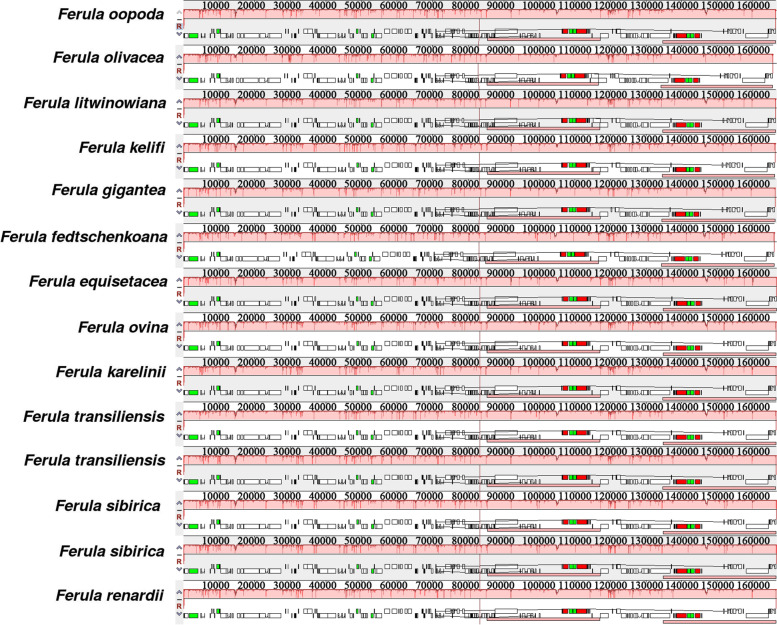
Sequence identity plots of the newly sequenced chloroplast genomes

**Fig. 5:**
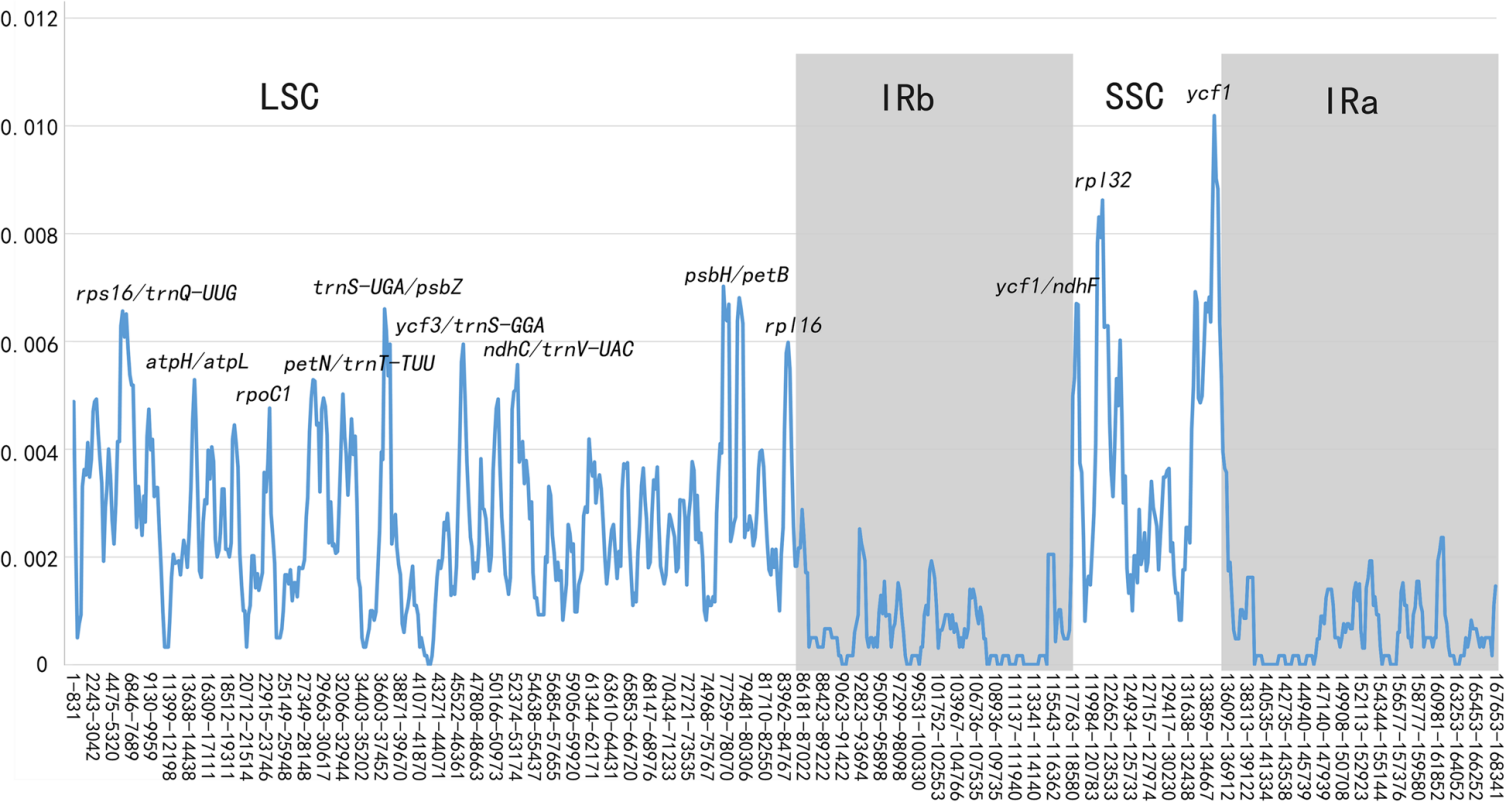
Sliding window analysis of the newly sequenced chloroplast genomes of *Ferula* species

According to the sequence identity plots, the 14 sequences were almost identical in their genetic structure and showed a very high degree of conservation (Fig. 4). To determine divergent hotspots, nucleotide diversity (Pi) values were calculated (Fig. 5, Table S5), yielding a maximum value of 0.01019 in *ycf1*. The SSC area showed the maximum nucleotide diversity followed by the LSC region, and the IR regions had the lowest Pi value. Additionally, six highly divergent regions (> 0.006) were detected in the LSC region (*rps16*/*trnQ-UUG*, *trnS-UGA*/*psbZ*, *psbH*/*petB*), SSC region (*ycf1*/*ndhF*, *rpl32*, *ycf1*), and IR region (0).

We calculated the Ka/Ks ratios of the 79 common protein-coding genes to reveal selection patterns among the protein-coding genes. The Ka/Ks ratios of most of the genes were less than 0.5 or could not be computed because either the Ka or Ks value was zero; three genes (*ccsA*, *ndhC*, and *ycf2*) had values greater than 1; and the total Ka/Ks ratio of all genes was 0.5331 (Table S6). In addition, we found several annotation errors (*ndhH* and *ccsA*) in the previously reported sequences of *F. sinkiangensis* (MW411057).

### Phylogenetic analyses

**Fig. 6:**
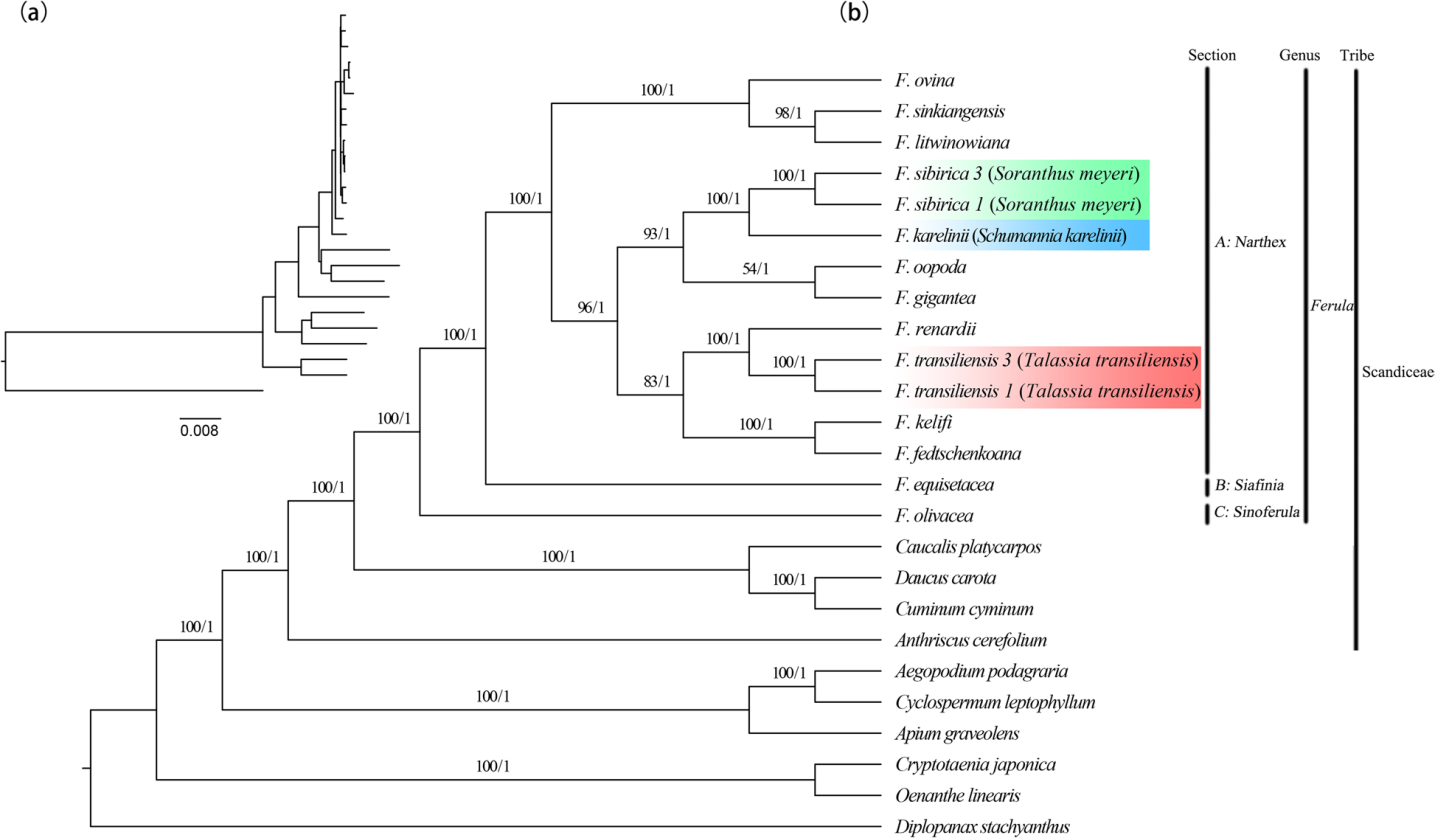
**a** Branch length diagram of the phylogenetic tree. **b** Phylogenetic tree of the 25 species inferred from maximum likelihood (ML) and Bayesian inference (BI) analyses based on the complete plastomes. The Shimodaira-Hasegawa-like support values approximate the likelihood ratio test (only *F. oopoda* and *F. gigantea* had SH-aLRT values below 80 in the terminal branch), and ultrafast bootstrap values (UFBS ≥ 95%, on the right) are shown on the branches. Green indicates two sequences of *S. meyeri* (*F. sibiraca*), blue indicates one sequence of *S. karelinii* (*F. karelinii*), and red indicates two sequences of *T. transiliensis* (*F. transiliensis*)

To determine the phylogenetic relationship of *Soranthus* Ledeb., *Schumannia* Kuntz., *Talassia* Korovin, and *Ferula* L., 25 chloroplast genomes were used to construct maximum likelihood (ML) and Bayesian inference (BI) phylogenetic trees. These included 10 samples of 10 *Ferula* species (including *F. sinkiangensis*, GenBank accession no. MW411057), two samples of *Soranthus*, two samples of *Schumannia*, one sample of *Talassia*, and nine other Apiaceae genera, i.e., *Caucalis* L., *Daucus* L., *Cuminum* L., *Anthriscus* Pers., *Aegopodium* L., *Cyclospermum* Lag., *Apium* L., *Cryptotaenia* DC., and *Oenanthe* L. with an outgroup of *Diplopanax stachyanthus* Hand.-Mazz (Fig. 6).

The ML and BI topologies were highly supported. Ten selected genera formed 10 monophyletic groups, all of which had support values of 100 or 1 in the ML and BI trees, respectively. *Ferula* was divided into three main lineages (A, B and C) with maximal support (PP = 1, BS ≥ 97%), and three genera (*Soranthus meyeri*, *Schumannia karelinii*, *Talassia transiliensis*) were clustered into *Ferula*. Lineage A contained 11 *Ferula* species, *S. meyeri*, *T. transiliensis*, and *S. karelinii*. Within this lineage, *S. sibirica* and *S. karelinii* are sister species, and *F. sinkiangensis* and *F. litwinowiana* are sister species. Lineages B and C contained only *F. equisetacea* and *F. olivacea*, respectively. Moreover, *Ferula* and four genera of Apiaceae formed a monophyletic group.

## Discussion

### Comparison of *Ferula* plastid genome

Plastomes are considered an effective means used in taxonomic and evolutionary studies to assess evolutionary relationships and compare genome structure at different taxonomic levels [34–42]. Generally, the plastomes are highly conserved in genome structure, gene order, and gene content [32]. In this study, all 14 plastomes are divided into four regions consisting of an LSC (84,839–85,598 bp), an SSC (17,559–17,687 bp), and two IRs (31,392–31,880 bp). The comparative analysis of 14 complete plastomes showed great similarities in terms of genome length (165,607–167,013 bp), structure, IR/SC borders and GC content (37.8–38.0), the equal number of CDs, rRNA, and tRNA genes, and no rearrangement or a good collinearity relationship among them (Fig. 1; Table S1), indicated that the *Ferula* are relatively conserved.

Although the IR region is thought to be the most conserved region in the chloroplast genome, contraction and expansion of the IR region is common, and is the main reason for the variation in chloroplast genome size [44–46]. The junction of IRb/LSC located at *ycf2* gene is defined as the type without any expansion or contraction [47]. In this study, we observed that 14 sequenced complete plastomes exhibited significant IR expansion (Fig. 3). All the species expanded into *rps19* at the IRb/LSC junction region, contributing to *rps19* fragment in the IRa/LSC region, and they also expanded into *ycf1* at the IRb/SSC junction region, leading to an overlap between the *ycf1* pseudo-gene and *ndhF*. This was consistent with previous studies, in which the pseudogenes *ycf1* and *rps19* were produced by contraction and expansion of the IR region in angiosperms [48–50].

RSCU value is the ratio of specific codon usage frequency to desired frequency, which can eradicate the influence of amino acid composition on codon usage and promotes the detection of synonymous codons [51, 52]. Generally, the content of A/T was higher than that of G/C in plastomes codons and A/T is preferred in the third codon position [53], the bias also showed in the *Ferula* plastomes (Fig. 2). Leucine was encoded by 6 codons, the order of codon preference was UTA > CUT > UTG > CUA > CUC > CUG, which following previous studies [54, 55]. The analysis of RSCU can provide a basis for studying the specific mechanism of synonymous codon bias preference in different species, which plays a crucial role in molecular biology basis research [56, 57].

As a primary source of molecular markers, SSRs have been widely used in *Ferula* genetic diversity studies because of their high polymorphism rate and abundant variation at the species level [58, 59]. In our study, we identified 837 repeats (Table S3) and 1,061 SSRs (Table S4) in the 14 *Ferula* samples. In which, the single nucleotide and dinucleotide repeats were common, which is consistent with the results of previous studies [55, 60]. In general, during the evolutionary process of species, most repeated sequences in the genome are distributed in the non-coding region and retain as little genetic information as possible to improve its genetic efficiency. Therefore, repeat sequences play an important role in species evolution [61–63]. The repeats found in the 12 analyzed species indicate genetic variation among the *Ferula* species. In addition, we also observed that the poly (A/T) SSRs were typically most common, while poly (C/G) repeats were extremely rare. These results are consistent with those of a previous study and verify the hypothesis that cpSSRs generally consist of short polyadenine (polyA) or polythymine (polyT) repeats and rarely contain tandem guanine (G) or cytosine (C) repeats [64–66].

Divergent hotspots play a significant role in species identification and phylogenetic information. Moreover, IR regions often show lower sequence divergence than SSC and LSC regions [67], this probably due to higher mutation rates lead to rapid genome evolution compared to other regions [68]. In our study, this phenomenon was evident that the SSC area showed the maximum nucleotide diversity followed by the LSC region, and the IR regions had the lowest Pi value (Fig. 5, Table S5). And *rps16*/*trnQ-UUG*, *trnS-UGA*/*psbZ*, *psbH*/*petB*, *ycf1*/*ndhF*, *rpl32*, *ycf1* were detected as the most divergent regions (Pi > 0.006) across all tested plastomes, suggesting that these variable loci can be used as important references and potential molecular markers for future studies on the evolution and diversity in *Ferula*. Generally, the Ka/Ks ratio is used to divide genes into positive selection, neutral evolution, and purification, with a limit of one [69]. Previously studies indicated that Ka/Ks ratios mostly are lower due to synonymous nucleotide substitutions rates that occur more often compared to nonsynonymous substitutions rates [70]. The genes with the highest Ka/Ks variability can be used as candidate barcodes to diferentiate species and in the future applied to perform phylogenetic and phylogeographic analyses [71]. Our study suggests that 76 common protein-coding genes were under purifying selection, which indicates the typical evolutionary conservation of plant plastid genes [55, 72, 73], and three genes (*ccsA*, *ndhC*, *ycf2*) were under weak positive selection (Table S6), *ycf2* have been proved to be pseudogenized in many studies [74] and *ccsA* was located in one of the most divergent regions, possibly as a discriminating DNA barcode for *Ferula* species.

### The relationships between *Soranthus*, *Schumannia*, *Talassia* and *Ferula*

Based on the anatomical morphological characteristics of sclerosing cell layers in the mesocarp, the genera *Soranthus*, *Schumannia* and *Talassia* have been proposed to be located under the genus *Ferula* [25, 75], all of which are recognized in the Flora of China [76]. It is easily distinguished *Ferula* from *Soranthus* and *Schumannia* by gross morphology and inflorescence structure, combined with the presence of luteolin 7-glycosides in the leaves, that seems reasonable to combine the two genera into *Soranthus* [77]. Also, *Talassia* tends to be incorporated into *Ferula* because insignificant morphological differences, although a large extent similarity between *T. transiliensis* and *F. conocaula* in the spectrum of leaf flavonoids [77]. Through a comparative study of plant external morphology, fruit anatomy, and pollen morphology, Qin and Shen [27] suggest that *Talassia* should be an independent genus and agreed to combine the other two monotypic genera. However, the above four genera have been suggested to merge into one genus according to the presence or absence of coumarins [78]. Recentlly, the molecular phylogeny of *Ferula* constructed Kurzyna-Młynik et al. [7] and Panahi et al. [16, 29] based on nrDNA ITS and cpDNA sequences (the *rps16* intron, the *rpoC1* intron and the *rpoB-trnC*) indicated that *Soranthus*, *Schumannia*, and *Talassia* were embedded in *Ferula* with low support values. In our study, 15 sequences (including *S. meyeri*, *S. karelinii* and *T. transiliensis*) covered all of the branches except the subgenera *Ferula* (including section *Ferula* and section *Stenocarpa*) according to the latest *Ferula* phylogenetic tree [16]. Our results show that all those three species representing the genera *Soranthus*, *Schumannia* and *Talassia* were embedded in *Ferula* based on phylogenetic trees with high bootstrap values (Fig. 6). The species *S. meyeri* and *S. karelinii* were clustered into section *Soranthus* (PP = 1, BS = 100%), and *T. transiliensis* and *F. renardii* clustered into section *Glaucoselinum* (PP = 1, BS = 100%), which was coincident with Panahi [16] while with higher support values. Therefore, we support the standpoint of sinking *Soranthus* Ledeb., *Schumannia* Kuntz., *Talassia* Korovin into synonymy of *Ferula* L.

### Plastomes might provide new insight on phylogenetic relationships in *Ferula*

As one of a complex taxonomic genus within Apiaceae, the system of *Ferula* is paid attention at the morphological and molecular levels [7, 9–11, 13–16]. All those efforts on taxonomic systems have contributed greatly to understanding of the genus *Ferula*. Kurzyna-Młynik et al. [7] published the first molecular phylogeny for *Ferula* to solve the relationship among *Dorema*, *Ferula* and *Leutea*, in which nrDNA ITS sequences were used to construct a phylogenetic tree revising *Dorema* and *Leutea* to *Ferula* and transferring *Ferula* to Scandiceae from Peucedaneae. Later, nrDNA ITS sequences and three fragments of cpDNA (the *rps16* intron, the *rpoC1* intron and the *rpoB-trnC*) were used to explore the relationship among the three genera, and it was found that *Dorema* was incorporated into *Ferula* and *Leutea* independently [16, 29]. Although these results provide an important foundation for the identification and classification of *Ferula* species, all previous studies have been based on relatively short sequences with low support values owing to the relatively limited number of nuclear/chloroplast genes. In addition, nrDNA and plastid DNA are highly incongruent, and intense reticulate evolution in *Ferula* means that proposing an unambiguous hierarchical classification system is almost impossible [16]. Furthermore, many species of *Ferula* have not been specifically addressed, and many only broadly grouped into branches.

Notably, studies based on plastomes can provide new insights into the phylogenetic relationships between species. For example, *Clerodendranthus spicatus* is closely related to two Lamiacea species, *Tectona grandis* L.f. and *Glechoma longituba* (Nakai) Kuprian. [79]; Juglandaceae is monophyletic, and *Carya cathayensis* Sarg. is a sister to *C. kweichowensis* Kuang & A.M.Lu and *C. illinoinensis (Wangenh.)* K.Koch [66]; and *Fagus longipetiolata* Seemen and *F. engleriana* Seemen ex Diels form a close relationship [41]. Here, we performed phylogenetic analyses for *Ferula* and other genera of Apiaceae using complete plastomes, and we recognized *Ferula* as a monophyletic group with the integration of *Soranthus*, *Schumannia*, *Talassia* (PP = 1, BS = 100%). Within *Ferula*, we recovered three main lineages in agreement with Panahi et al. [16], who proposed a new classification based on morphological characteristics and sequence data (nrDNA ITS sequences and three cpDNA fragments). This classification divides *Ferula* into four subgenera and 10 sections. In addition, *Caucalis*, *Daucus*, *Cuminum*, and *Anthriscus* were all typical of Scandiceae and formed a monophyletic system with *Ferula*. This provides strong evidence and support for the transfer of *Ferula* from the Peucedaneae to the Scandiceae [7]. However, we also observed some differences. When added into Panahi et al.’s phylogenetic tree, *F. sinkiangensis* was clustered into the *Scorodosma* branch with the sister species *F. kelifi*. Based on our results, *F. sinkiangensis* is separated from *F. kelifi*, being clustered with *F. litwinowiana* in the *Merwia* branch. Further research is needed to confirm this phenomenon. Overall, our work demonstrates that plastome studies can provide highly useful information for future phylogenetic, taxonomic, and evolutionary studies of *Ferula*.

## Conclusion

We obtained 14 complete cp genome sequences from 12 *Ferula* species (including *Soranthus*, *Schumannia*, and *Talassia*) and compared them based on genome structure, gene content, and gene sequences. Some hotspots in the LSC and SSC regions were identified, which may provide useful markers for phylogenetic analysis. Notably, the Gene *ccsA* can be used as a DNA barcode for *Ferula* species. Our phylogenetic analysis showed a tight connection between *Soranthus* Ledb., *Schumannia* Kuntz., *Talassia* Korov., and *Ferula* L., indicating that treatment as separate genera is unreasonable. Instead, their phylogenetic relationship, which is now well resolved, strongly supports that they can be considered synonymous with *Ferula*. This new genomic information not only contributes to the better development and utilization of *Ferula* but also provides a basis for further understanding the evolutionary, genetic, and phylogenetic relationships of this important genera.

## Materials and methods

### Plant materials and DNA extraction

Fourteen samples were collected from the field and herbaria (Table S1). Of these, five specimens were taken from the specimen museum of the Xinjiang Institute of Ecology and Geography, Chinese Academy of Sciences (XJBI), one was obtained from the Komarov Botanical Institute of RAS (LE), five specimens were taken from the National Herbarium of Uzbekistan (TASH), and three were collected from the field in Tajikistan. Leaf samples were dried in silica gel and stored at -20 °C for DNA extraction. DNA extraction was performed using a plant genome extraction kit (DP320) from Tiangen Biochemical Technology (Beijing) according to the manufacturer’s instructions.

### DNA sequencing and genome assembly and annotation

The extracted DNA was sent to a sequencing company for automatic sequencing using the NEBNext Ultra II DNA Library Prep Kit for Illumina (New England BIolabs) [80]. DNA extracts were quantified and sheared into approximately 500 base pair (bp) fragments for library construction using standard protocols (NEBNext Ultra IITMDNA Library Prep Kit for Illumina). Paired-end sequencing from both ends of 150 bp fragments was performed on the Illumina HiSeq X Ten platform at the Molecular Biology Experiment Center, Germplasm Bank of Wild Species in Southwest China, to generate no less than 2 GB data for each individual.

The paired-end reads were filtered using the GetOrganelle pipeline (https://github.com/Kinggerm/GetOrganelle) to obtain plastid-like reads [81] and then assembled using SPAdes version 3.10 [82]. A complete circular assembly graph was checked and further extracted using Bandage version 0.8.1 [83]. The genomes were automatically annotated using CpGAVAS [84], PGA (https://github.com/quxiaojian/PGA), and then manually adjusted using Geneious version 9.1.7 [85]. The chloroplast sequences generated in this study have been submitted to GenBank (Table S1). Circular genome maps of all 14 plastomes were also obtained using the Organellar Genome DRAW (OGDRAW) tool [86].

### Codons, repeat sequences, and simple sequences repeat analysis

The protein-coding genes were extracted for codon analysis. The final dataset included 86 protein-coding genes from each species. Codon usage and relative synonymous codon usage (RSCU) values were calculated using JSHYCloud (http://cloud.genepioneer.com:9929). A heatmap of all the RSCU values of the 14 plastomes was produced using ClustVis [87]. Using the parameters of a Hamming distance of 3, a minimum repeat size of 30 bp, and a maximum repeat size of 5,000 bp, REPuter was used to identify the size and location of four types of repeat sequences (i.e., forward, palindromic, reverse, and complement) [88]. Simple sequence repeats (SSRs) were detected using the online MISA software (http://pgrc.ipkgatersleben.de/misa/misa.html) with minimum repeat number settings of 10, 5, 4, 3, 3, and 3 for mononucleotides, dinucleotides, trinucleotides, tetranucleotides, pentanucleotides, and hexanucleotides, respectively.

### Genome comparison with other *Ferula* species and selective pressure analysis

Sequence divergence among the 14 chloroplast (cp) genomes was compared using Mafft (version 7.0) [89], IRscope (https://irscope.shinyapps.io/irapp/) and Mauve [90]. DnaSP [91] was used to calculate nucleotide divergence values using the sliding window method, with a window length of 800 bp and a step size of 200 bp. Selective pressures were analyzed for 79 common protein-coding genes among 15 *Ferula* species (including one published plastome). The ratio of nonsynonymous to synonymous nucleotide substitution rates (Ka/Ks) was calculated using DnaSP.

### Phylogenetic analysis

We used 25 complete plastome sequences to infer the phylogenetic relationships of *Ferula*. After comparison with Mafft, Trimal [92], and Phylosuite [93] were used to trim areas with poor quality. The phylogenetic tree was then constructed using RaxML-HPC v.8 [94] and the maximum likelihood method with 1,000 replicates and the GTRGAMMA model. After screening for the best model using jModelTest2 [95], MrBayes 3.2.7a [96] was used to construct a Bayes tree, and the selected models for the complete plastome sequences in BI analyses were TPM1uf + I + G, and iTOL [97] and FigTree 1.4.2 [98] were used to construct the phylogenetic tree.

## Supplementary Information

**Additional file 1:** **Table S1.** All the materials used in this article.

**Additional file 2:** **Table S2.** Codon usage and relative synonymous codon usage (RSCU) values of protein-coding genes of the 14 Ferula plastomes.

**Additional file 3:** **Table S3.** Distribution of repeat sequences in the 14 studied Ferula plastomes.

**Additional file 4:** **Table S4.** Distribution of simple sequence repeats (SSRs) in the 14 studied Ferula plastomes.

**Additional file 5:** **Table S5.** Nucleotide variability (Pi) of Ferula species.

**Additional file 6:** **Table S6.** Non-synonymous to synonymous nucleotide substitution rates (Ka/Ks) of Ferula species.

## Data Availability

Fourteen newly sequenced and annotated plastomes have been submitted into NCBI (https://www.ncbi.nlm.nih.gov). Accession numbers: ON324036-ON324048, OM993535.

## References

[1] Pimenov MG, Leonov MV (1993). The genera of the Umbelliferae: a nomenclator.

[2] Sina AA. Kanon vrachebnoy nauki (Canon). In: Zaxidov TZ, editor. Book. 1. Tashkent: Academy of Sciences of the UzSSR; 1954. p. 549.

[3] Shen GM (1986). Chinese herbal medicine series: Ferula.

[4] Nazari ZE, Iranshahi M (2011). Biologically active sesquiterpene coumarins from Ferula species. Phytother Res.

[5] Mahendra P, Bisht S (2012). Ferula asafoetida: Traditional uses and pharmacological activity. Pharmacogn Rev.

[6] Ajani Y, Ajani A, Cordes JM, Watson MF, Downie SR (2008). Phylogenetic analysis of nrDNA ITS sequences reveals relationships within five groups of Iranian Apiaceae subfamily Apioideae. Taxon.

[7] Kurzyna-Młynik R, Oskolski AA, Downie SR, Kopacz R, Wojewódzka A, Spalik K (2008). Phylogenetic position of the genus Ferula (Apiaceae) and its placement in tribe Scandiceae as inferred from nrDNA ITS sequence variation. Plant Syst Evol.

[8] Safina LK, Ostroumova TA, Pimenov MG (2015). Carpology of the species of Ferula subgen. Merwia(Umbelliferae-Apioideae) and some taxonomic implications. Nord J Bot.

[9] Boissier PE. Flora orientalis sive, enumeratio plantarum in Oriente a Graecia et Aegypto ad Indiae fines hucusque observatae 2. Genève, Basel & Lyon: H.Georg; 1872. p. 1159.

[10] Drude CGO. Umbelliferae. In: Engler A, Prantl K, editors. Die natürlichen Pflanzenfamilien, vol. 3. Leipzig: Verlag von Wilhelm Engelman (Druck von Breitkopf & Härtel in Leipzig); 1898. p. 63–250.

[11] Korovin EP. Generis Ferula (Tourn.) L. monographia illustrata. Tashkent: Academiae Scientiarum UzRSS; 1947. p. 91.

[12] Chamberlain DF, Rechinger KH. Ferula L. In: Hedge IC, Lamond JM, Rechinger KH, editors. Umbelliferae, Flora Iranica, vol. 162. Graz: Akademische Druck- und Verlagsanstalt; 1987. p. 387–426.

[13] Safina LK, Pimenov MG (1983). The carpoanatomical features of the species of the genus Ferula of the subgenus Peucedanoides (Apiaceae) in connection with the systematics of the genus. Bot Zhurn (Leningrad).

[14] Safina LK, Pimenov MG. Feruly Kazakhstana. Alma-ata: Nauka Kazakhskoĭ SSR; 1984. p. 110.

[15] Safina LK, Pimenov MG (2008). Carpology of the species of type subgenus of the genus Ferula and some problems of their systematics. Feddes Repertorium.

[16] Panahi M, Banasiak L, Piwczyński M, Puchałka R, Kanani MR, Oskolski AA, Modnicki D, Miłobędzka A, Spalik K (2018). Taxonomy of the traditional medicinal plant genus Ferula (Apiaceae) is confounded by incongruence between nuclear rDNA and plastid DNA. Bot J Linn Soc.

[17] Ledebour CF, Bunge A, Meyer CA. Flora Altaica. Berolini: G. Reimeri; 1829. p. 197–206.

[18] von Bunge A. Beitrag zur kenntniss der flor Russlands und der steppen Central-Asiens. St. Petersburg: Kaiserliche Akademie der Wissenschaften; 1851. p. 359.

[19] Korovin EP, Schischkin BK (1951). Ferula L. Flora of the USSR.

[20] She ML, Pu FD, Pan ZH, Watson MF, Cannon JFM, Holmes-Smith I, et al. Apiaceae (Umbelliferae). In: Wu ZY, Raven RH, editors. Flora of China. Beijing and St. Louis: Science Press and Missouri Botanical garden Press; 2005. p. 1–205.

[21] Shen GM. Apiaceae (Umbelliferae). In: Shen GM, editor. Flora Xinjiangensis. Urumqi: Xinjiang Science & Technology Publishing House; 2011. p. 464–621.

[22] Kuntze O. Plantae orientali-rossicae. Trudy Imp: S.-Peterburgsk. Bot. Sada. 1887;10:35–262.

[23] Tojibaev KSh, Sennikov AN, Lazkov GA, Jang GG, Choi HJ, Chang KS, et al. Checklist of vascular plants of the Tian-Shan Mountain System. Pocheon: Korea National Arboretum; 2021. p. 607.

[24] Korovin EP. The new genera and species of Umbelliferae from Kazakhstan flora. Trudy Instituta Botaniki: Akademiya Nauk Kazakhskoi SSR. 1962;13:242–62.

[25] Pimenov MG, Kirillina NA (1980). The carpology of Soranthus, Ladyginia, Eriosynaphe and Schumannia in connection with the problem of the taxonomic limits of the genus Ferula (Apiaceae). Botanicheskii Zhurnal.

[26] Govaerts R, Nic Lughadha E, Black N, Turner R, Paton A (2021). The World Checklist of Vascular Plants, a continuously updated resource for exploring global plant diversity. Sci Data.

[27] Qin XM, Shen KM (1990). Taxonomic studies on the Genus Ferula and its close genera in Xinjiang. Arid Zone Res.

[28] Hui H, Liu QX, Liu MH (2003). Study on serum classification and genetic relationship of Ferula of Peucedaneae subtribe Ferulinae of Apiaceae in China. J Syst Evol.

[29] Panahi M, Banasiak Ł, Piwczyński M, Puchałka R, Oskolski AA, Spalik K (2015). Phylogenetic relationships among Dorema, Ferula and Leutea (Apiaceae: Scandiceae: Ferulinae) inferred from nrDNA ITS and cpDNA noncoding sequences. Taxon.

[30] Corriveau JL, Coleman AW (1988). Rapid Screening Method to Detect Potential Biparental Inheritance of Plastid DNA and Results for over 200 Angiosperm Species. Am J Bot.

[31] Jansen RK, Raubeson LA, Boore JL, dePamphilis CW, Chumley TW, Haberle RC, Wyman SK, Alverson AJ, Peery R, Herman SJ (2005). Methods for obtaining and analyzing whole chloroplast genome sequences. Methods Enzymol.

[32] Ravi V, Khurana JP, Tyagi AK, Khurana P (2007). An update on chloroplast genomes. Plant Syst Evol.

[33] Wicke S, Schneeweiss GM, dePamphilis CW, Muller KF, Quandt D (2011). The evolution of the plastid chromosome in land plants: gene content, gene order, gene function. Plant Mol Biol.

[34] Yang JB, Tang M, Li HT, Zhang ZR, Li DZ (2013). Complete chloroplast genome of the genus Cymbidium: lights into the species identification, phylogenetic implications and population genetic analyses. BMC Evol Biol.

[35] Dong W, Liu H, Xu C, Zuo Y, Chen Z, Zhou S (2014). A chloroplast genomic strategy for designing taxon specific DNA mini-barcodes: a case study on ginsengs. BMC Genet.

[36] Ma PF, Zhang YX, Zeng CX, Guo ZH, Li DZ (2014). Chloroplast phylogenomic analyses resolve deep-level relationships of an intractable bamboo tribe Arundinarieae (poaceae). Syst Biol.

[37] Coissac E, Hollingsworth PM, Lavergne S, Taberlet P (2016). From barcodes to genomes: extending the concept of DNA barcoding. Mol Ecol.

[38] Hollingsworth PM, Li DZ, van der Bank M, Twyford AD (2016). Telling plant species apart with DNA: from barcodes to genomes. Philos Trans R Soc Lond B Biol Sci.

[39] Huang Y, Li X, Yang Z, Yang C, Yang J, Ji Y (2016). Analysis of Complete Chloroplast Genome Sequences Improves Phylogenetic Resolution in Paris (Melanthiaceae). Front Plant Sci.

[40] Xie DF, Yu Y, Deng YQ, Li J, Liu HY, Zhou SD, He XJ (2018). Comparative Analysis of the Chloroplast Genomes of the Chinese Endemic Genus Urophysa and Their Contribution to Chloroplast Phylogeny and Adaptive Evolution. Int J Mol Sci.

[41] Liang D, Wang H, Zhang J, Zhao Y, Wu F (2022). Complete Chloroplast Genome Sequence of Fagus longipetiolata Seemen (Fagaceae): Genome Structure, Adaptive Evolution, and Phylogenetic Relationships. Life (Basel).

[42] Wang N, Chen S, Xie L, Wang L, Feng Y, Lv T, Fang Y, Ding H (2022). The complete chloroplast genomes of three Hamamelidaceae species: Comparative and phylogenetic analyses. Ecol Evol.

[43] Fu CN, Mo ZQ, Yang JB, Cai J, Ye LJ, Zou JY, Qin HT, Zheng W, Hollingsworth PM, Li DZ (2022). Testing genome skimming for species discrimination in the large and taxonomically difficult genus Rhododendro. Mol Ecol Resour.

[44] Goulding SE, Olmstead RG, Morden CW, Wolfe KH (1996). Ebb and flow of the chloroplast inverted repeat. Mol Gen Genet.

[45] Huang J-L, Sun G-L, Zhang D-M (2010). Molecular evolution and phylogeny of the angiosperm ycf2 gene. J Syst Evol.

[46] Zhu A, Guo W, Gupta S, Fan W, Mower JP (2016). Evolutionary dynamics of the plastid inverted repeat: the effects of expansion, contraction, and loss on substitution rates. New Phytol.

[47] Wen J, Xie DF, Price M, Ren T, Deng YQ, Gui LJ, Guo XL, He XJ (2021). Backbone phylogeny and evolution of Apioideae (Apiaceae): New insights from phylogenomic analyses of plastome data. Mol Phylogenet Evol.

[48] Gu C, Ma L, Wu Z, Chen K, Wang Y (2019). Comparative analyses of chloroplast genomes from 22 Lythraceae species: inferences for phylogenetic relationships and genome evolution within Myrtales. BMC Plant Biol.

[49] Liu X, Chang E-M, Liu J-F, Huang Y-N, Wang Y, Yao N, Jiang Z-P (2019). Complete Chloroplast Genome Sequence and Phylogenetic Analysis of Quercus bawanglingensis Huang, Li et Xing, a Vulnerable Oak Tree in China. Forests.

[50] Kim S-C, Lee J-W, Choi B-K (2021). Seven Complete Chloroplast Genomes from Symplocos: Genome Organization and Comparative Analysis. Forests.

[51] Rono PC, Dong X, Yang JX, Mutie FM, Oulo MA, Malombe I, Kirika PM, Hu GW, Wang QF (2020). Initial Complete Chloroplast Genomes of Alchemilla (Rosaceae): Comparative Analysis and Phylogenetic Relationships. Front Genet.

[52] Wanga VO, Dong X, Oulo MA, Mkala EM, Yang JX, Onjalalaina GE, Gichua MK, Kirika PM, Gituru RW, Hu GW (2021). Complete Chloroplast Genomes of Acanthochlamys bracteata (China) and Xerophyta (Africa) (Velloziaceae): Comparative Genomics and Phylogenomic Placement. Front Plant Sci.

[53] Morton BR (1998). Selection on the codon bias of chloroplast and cyanelle genes in different plant and algal lineages. J Mol Evol.

[54] Guisinger MM, Kuehl JV, Boore JL, Jansen RK (2011). Extreme reconfiguration of plastid genomes in the angiosperm family Geraniaceae: rearrangements, repeats, and codon usage. Mol Biol Evol.

[55] Ren T, Li ZX, Xie DF, Gui LJ, Peng C, Wen J, He XJ (2020). Plastomes of eight Ligusticum species: characterization, genome evolution, and phylogenetic relationships. BMC Plant Biol.

[56] Lu H, Zhao WM, Zheng Y, Wang H, Qi M, Yu XP (2005). Analysis of synonymous codon usage bias in Chlamydia. Acta Biochim Biophys Sin (Shanghai).

[57] Hassan S, Mahalingam V, Kumar V (2009). Synonymous codon usage analysis of thirty two mycobacteriophage genomes. Adv Bioinformatics.

[58] Li WJ, Su ZH, Yang L, Cao QM, Fengi Y (2020). Genetic diversity of the critically endangered Ferula sinkiangensis KM Shen (Apiaceae) and the implications for conservation. Turk J Bot.

[59] Yang L, Hisoriev H, Kurbonova P, Boboev M, Bobokalonov K, Feng Y, Li W (2021). High genetic diversity and low differentiation of endangered Ferula tadshikorum Pimenov in Tajikistan. GECCO.

[60] Ren T, Yang Y, Zhou T, Liu ZL (2018). Comparative Plastid Genomes of Primula Species: Sequence Divergence and Phylogenetic Relationships. Int J Mol Sci.

[61] Li B, Zheng Y (2018). Dynamic evolution and phylogenomic analysis of the chloroplast genome in Schisandraceae. Sci Rep.

[62] Chen Y, Hu N, Wu H (2019). Analyzing and Characterizing the Chloroplast Genome of Salix wilsonii. Biomed Res Int.

[63] Khan A, Asaf S, Khan AL, Al-Harrasi A, Al-Sudairy O, AbdulKareem NM, Khan A, Shehzad T, Alsaady N, Al-Lawati A (2019). First complete chloroplast genomics and comparative phylogenetic analysis of Commiphora gileadensis and C foliacea: Myrrh producing trees. PLoS One.

[64] Wang L, Wuyun T-n, Du H, Wang D, Cao D (2016). Complete chloroplast genome sequences of Eucommia ulmoides: genome structure and evolution. Tree Genet Genomes.

[65] Shen X, Wu M, Liao B, Liu Z, Bai R, Xiao S, Li X, Zhang B, Xu J, Chen S (2017). Complete Chloroplast Genome Sequence and Phylogenetic Analysis of the Medicinal Plant Artemisia annua. Molecules.

[66] Shen J, Li X, Chen X, Huang X, Jin S (2022). The Complete Chloroplast Genome of Carya cathayensis and Phylogenetic Analysis. Genes (Basel).

[67] Li W, Zhang C, Guo X, Liu Q, Wang K (2019). Complete chloroplast genome of Camellia japonica genome structures, comparative and phylogenetic analysis. PLoS ONE.

[68] Tyagi S, Jung JA, Kim JS, Won SY (2020). A comparative analysis of the complete chloroplast genomes of three Chrysanthemum boreale strains. PeerJ.

[69] Hurst LD (2002). The Ka/Ks ratio: diagnosing the form of sequence evolution. Trends Genet.

[70] Yang J, Kang GH, Pak JH, Kim SC (2020). Characterization and Comparison of Two Complete Plastomes of Rosaceae Species (Potentilla dickinsii var. glabrata and Spiraea insularis) Endemic to Ulleung Island, Korea. Int J Mol Sci.

[71] Dong X, Mkala EM, Mutinda ES, Yang JX, Wanga VO, Oulo MA, Onjolo VO, Hu GW, Wang QF (2022). Taxonomy, comparative genomics of Mullein (Verbascum, Scrophulariaceae), with implications for the evolution of Verbascum and Lamiales. BMC Genomics.

[72] Lee-Yaw JA, Grassa CJ, Joly S, Andrew RL, Rieseberg LH (2019). An evaluation of alternative explanations for widespread cytonuclear discordance in annual sunflowers (Helianthus). New Phytol.

[73] Zhang X, Deng T, Moore MJ, Ji Y, Lin N, Zhang H, Meng A, Wang H, Sun Y, Sun H (2019). Plastome phylogenomics of Saussurea (Asteraceae: Cardueae). BMC Plant Biol.

[74] Timme RE, Kuehl JV, Boore JL, Jansen RK. A comparison of the first two sequenced chloroplast genomes in Asteraceae: lettuce and sunflower. United States: Lawrence Berkeley National Laboratory; 2006. p. 1–33.

[75] Pimenov MG. Glaucoselinum section (Schischk.) M. Pimen of genus Ferula L. (Umbelliferae). Moscow, Biologicheskie nauki: Nauchnye doklady vysshei shkoly. 1983;12:74–9.

[76] Shan RH, She ML (1979). Flora Reipublcae Popularis Sinicae.

[77] Chen XY, Liu QX (1989). Luteolin glycosides as taxonomic markers in Ferula and related genera. Biochem Syst Ecol.

[78] Liu QX, Wu MY, Rao GX, Ye JS, Hui H (1999). H-NMR detection of coumarin and its application in the chemical classification of Ferula. J Plant Resour Environ.

[79] Du Q, Jiang M, Sun S, Wang L, Liu S, Jiang C, Gao H, Chen H, Li Y, Wang B (2022). The complete chloroplast genome sequence of Clerodendranthus spicatus, a medicinal plant for preventing and treating kidney diseases from Lamiaceae family. Mol Biol Rep.

[80] Meyer M, Kircher M (2010). Illumina sequencing library preparation for highly multiplexed target capture and sequencing. Cold Spring Harb Protoc.

[81] Jin JJ, Yu WB, Yang JB, Song Y, dePamphilis CW, Yi TS, Li DZ (2020). GetOrganelle: a fast and versatile toolkit for accurate de novo assembly of organelle genomes. Genome Biol.

[82] Bankevich A, Nurk S, Antipov D, Gurevich AA, Dvorkin M, Kulikov AS, Lesin VM, Nikolenko SI, Pham S, Prjibelski AD (2012). SPAdes: a new genome assembly algorithm and its applications to single-cell sequencing. J Comput Biol.

[83] Wick RR, Schultz MB, Zobel J, Holt KE (2015). Bandage: interactive visualization of de novo genome assemblies. Bioinformatics.

[84] Liu C, Shi L, Zhu Y, Chen H, Zhang J, Lin X, Guan X (2012). CpGAVAS, an integrated web server for the annotation, visualization, analysis, and GenBank submission of completely sequenced chloroplast genome sequences. BMC Genomics.

[85] Kearse M, Moir R, Wilson A, Stones-Havas S, Cheung M, Sturrock S, Buxton S, Cooper A, Markowitz S, Duran C (2012). Geneious Basic: an integrated and extendable desktop software platform for the organization and analysis of sequence data. Bioinformatics.

[86] Lohse M, Drechsel O, Kahlau S, Bock R (2013). OrganellarGenomeDRAW–a suite of tools for generating physical maps of plastid and mitochondrial genomes and visualizing expression data sets. Nucleic Acids Res.

[87] Metsalu T, Vilo J (2015). ClustVis: a web tool for visualizing clustering of multivariate data using Principal Component Analysis and heatmap. Nucleic Acids Res.

[88] Kurtz S, Choudhuri JV, Ohlebusch E, Schleiermacher C, Stoye J, Giegerich R (2001). REPuter: the manifold applications of repeat analysis on a genomic scale. Nucleic Acids Res.

[89] Katoh K, Rozewicki J, Yamada KD (2019). MAFFT online service: multiple sequence alignment, interactive sequence choice and visualization. Brief Bioinform.

[90] Darling AC, Mau B, Blattner FR, Perna NT (2004). Mauve: multiple alignment of conserved genomic sequence with rearrangements. Genome Res.

[91] Rozas J, Ferrer-Mata A, Sanchez-DelBarrio JC, Guirao-Rico S, Librado P, Ramos-Onsins SE, Sanchez-Gracia A (2017). DnaSP 6: DNA Sequence Polymorphism Analysis of Large Data Sets. Mol Biol Evol.

[92] Capella-Gutierrez S, Silla-Martinez JM, Gabaldon T (2009). trimAl: a tool for automated alignment trimming in large-scale phylogenetic analyses. Bioinformatics.

[93] Zhang D, Gao F, Jakovlic I, Zou H, Zhang J, Li WX, Wang GT (2020). PhyloSuite: An integrated and scalable desktop platform for streamlined molecular sequence data management and evolutionary phylogenetics studies. Mol Ecol Resour.

[94] Stamatakis A (2014). RAxML version 8: a tool for phylogenetic analysis and post-analysis of large phylogenies. Bioinformatics.

[95] Darriba D, Taboada GL, Doallo R, Posada D (2012). jModelTest 2: more models, new heuristics and parallel computing. Nat Methods.

[96] Ronquist F, Teslenko M, van der Mark P, Ayres DL, Darling A, Hohna S, Larget B, Liu L, Suchard MA, Huelsenbeck JP (2012). MrBayes 32: efficient Bayesian phylogenetic inference and model choice across a large model space. Syst Biol.

[97] Letunic I, Bork P (2021). Interactive Tree Of Life (iTOL) v5: an online tool for phylogenetic tree display and annotation. Nucleic Acids Res.

[98] Rambaut A. FigTree 1.4.2 software, a graphical viewer of phylogenetic trees. Edinburgh: Institute of Evolutionary Biology University of Edinburgh; 2014.

